# An Unusual Case of Bilateral Ureteric Obstruction Caused by Colorectal Cancer

**DOI:** 10.7759/cureus.87132

**Published:** 2025-07-01

**Authors:** Raamish Asad Raza, Muhammad Mairaj Uddin Siddiqui, Tariq Aziz, Sayyeda Niha Akhtar

**Affiliations:** 1 Emergency Medicine, University Hospitals Dorset NHS Foundation Trust, Poole, GBR; 2 Pediatrics, Usman Memorial Hospital, Karachi, PAK

**Keywords:** bilateral nephrostomy, bilateral ureteric obstruction, colorectal cancer, decrease urine output, hyperkalaemia, jj stent, upper urinary tract obstruction, ureteric obstruction, urinary system obstruction

## Abstract

Acute kidney injury (AKI) is a common complication in patients with cancer. The aetiology of these cases can be categorized into pre-renal, intra-renal, and post-renal causes based on the location of the lesion. Post-renal AKI is caused by obstructive uropathy, especially in malignancies of the bladder, prostate, uterus, and cervix, but less commonly in colorectal cancer. This can eventually lead to hyperkalaemia, metabolic acidosis, and acute renal failure.

To emphasize that colorectal carcinoma is an uncommon cause of bilateral obstructive uropathy, we present this case of post-renal acute kidney injury to draw attention to and familiarize clinicians with its uncommon presentation.

An 81-year-old male patient with a past medical history of recurrent colorectal cancer presented with fatigue and low urine output for two days in the emergency department. His examination was unremarkable. His blood gas analysis showed metabolic acidosis, hyperkalaemia, and AKI (warning stage 3). A computed tomography (CT) scan showed new bilateral renal pelvicalyceal dilatation and ureteric obstruction. He was admitted to the intensive treatment unit (ITU) with a diagnosis of AKI secondary to bilateral ureteric obstruction due to relapse of his colorectal cancer. Bilateral nephrostomies were performed, and his blood parameters improved gradually, along with returning urine output.

## Introduction

Acute kidney injury (AKI) is defined as a sudden decline in kidney function characterized by a serum creatinine rise of ≥26.5 µmol/L in 48 hours or an elevation of 1.5 times over a weekly period [1]. AKI is classified into pre-renal, intra-renal, and post-renal AKI [1, 2]. Post-renal AKI is caused by either bilateral obstruction of the ureters, obstruction of a ureter in a person with a solitary kidney, or if the urinary tract below the bladder becomes obstructed [2].

This article focuses on obstructive uropathy, which is generally defined as the structural or functional obstruction to urinary flow, causing kidney damage [3]. Urinary obstruction has a bimodal distribution, with both infants and the elderly being affected [4]. Ureteric obstruction can lead to metabolic acidosis, hyperkalaemia, and post-renal AKI and is responsible for 5-10% of all renal injuries [5].

There are several causes of obstructive uropathy, with benign prostatic hyperplasia (BPH) considered the most common cause [3]. Other causes include prostate, cervical, ovarian, and colon cancer, retroperitoneal fibrosis, kidney stones, and papillary necrosis [4-6]. In a case series consisting of 50 cases of bilateral ureteric obstruction, 38 were linked to malignant causes, with only five cases associated with colon cancer, highlighting its rarity as a cause of this condition [6]. This case report discusses colorectal cancer causing a bilateral ureteric obstruction that led to post-renal AKI.

Colorectal cancer is the third most common cancer worldwide, with lung cancer being the most common. It is the second most common cause of cancer in the female population, while in males it remains the third most common. It also accounts for the second-highest cancer-related deaths in the world, just trailing behind lung cancer [7].

Treatment of ureteric obstruction consists of correcting the metabolic disturbances that develop, such as metabolic acidosis and hyperkalaemia. Renal replacement therapy (RRT) is initiated if the conservative treatment fails. The definitive treatment is to drain the blocked urinary tract system [1, 5].

The methods of drainage are controversial and remain a topic of debate. Percutaneous nephrostomy or placement of ureteral stents are the two most frequently used methods, with the latter being the most commonly recommended, especially in those with advanced disease. There has been a significant amount of relevant literature regarding these two methods, but no consensus has yet been reached [1]. Hence, choosing which method to use should be based on the patient’s needs and the doctor’s preference [1].

To highlight colorectal carcinoma as an uncommon cause of bilateral ureteric obstruction, we present the case of an 81-year-old male patient who developed bilateral ureteric obstruction caused by recurrence of his colorectal carcinoma that led to post-renal AKI and hyperkalaemia.

## Case presentation

**Figure 1:**
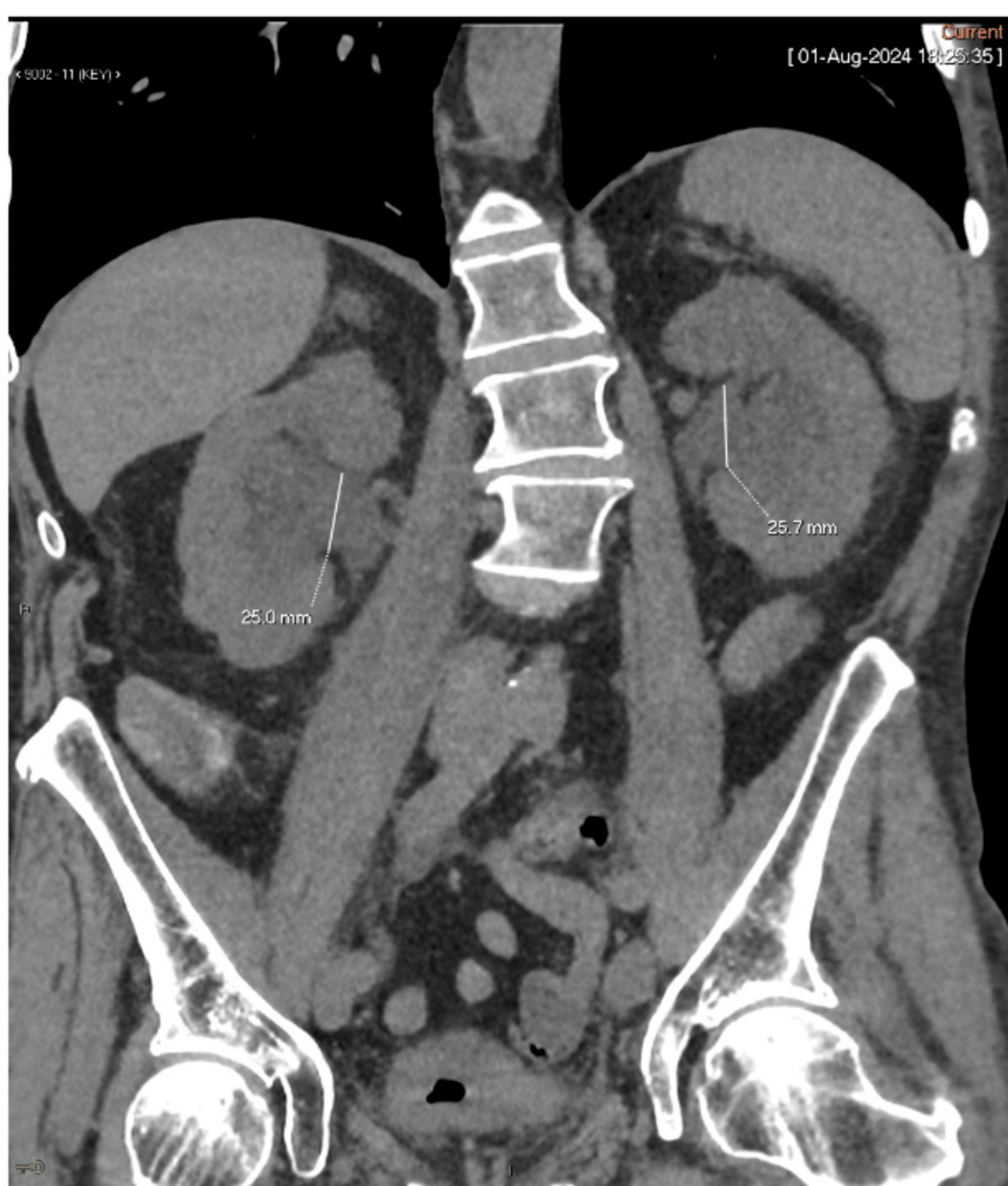
Coronal view of the CT scan of the abdomen and pelvis showing bilateral hydronephrosis

**Figure 2:**
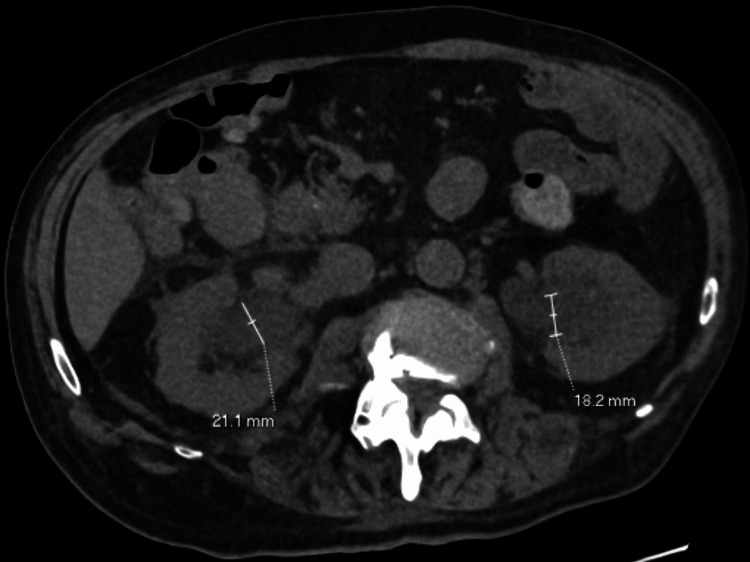
Axial view of the CT scan of the abdomen and pelvis showing moderate to severe bilateral hydronephrosis

**Figure 3:**
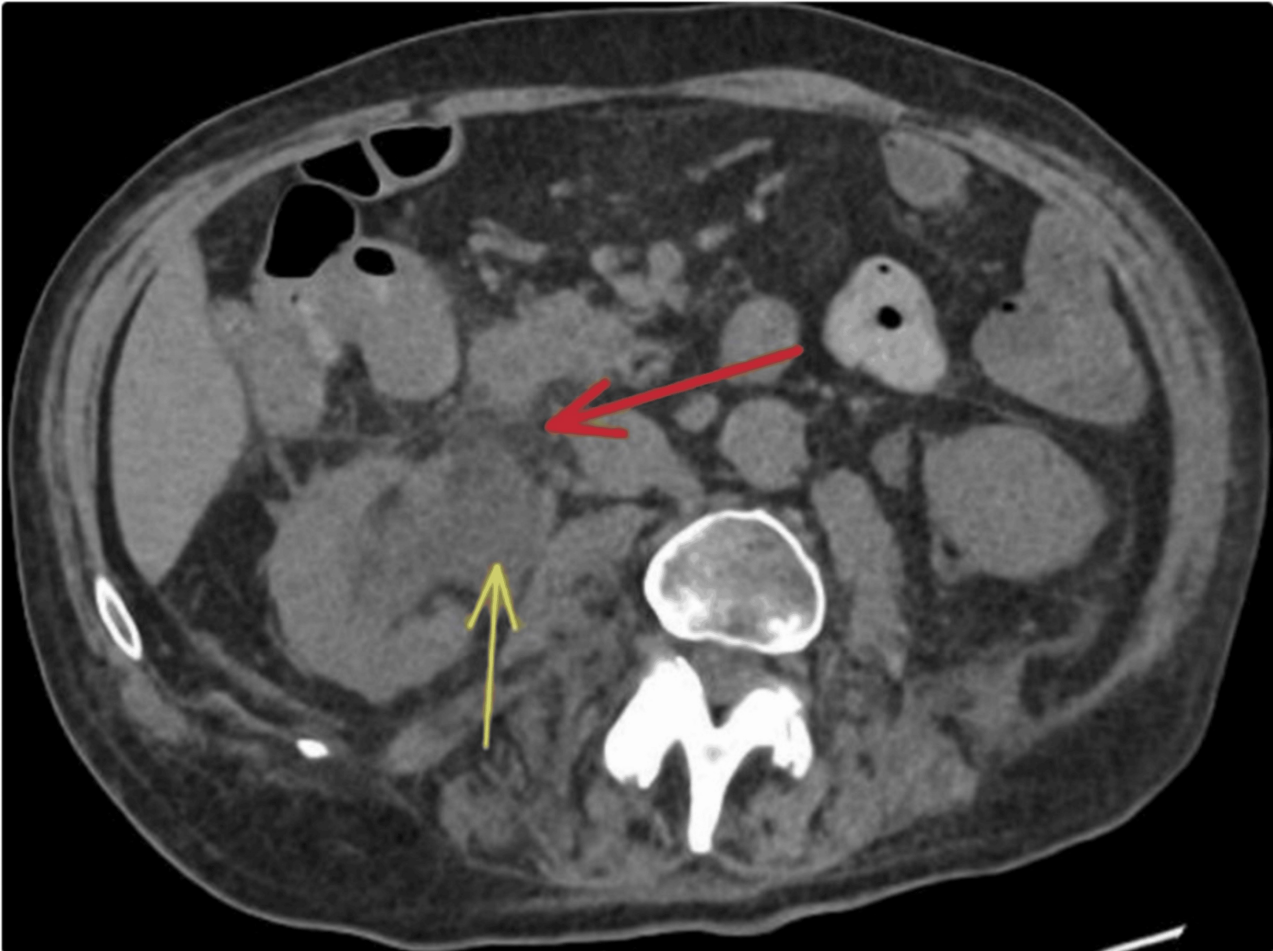
Axial view of the CT scan of the abdomen and pelvis. The red arrow points to the retroperitoneal fat stranding, and the yellow arrow points to the resulting right pelvicalyceal dilatation.

An 81-year-old male patient presented to the accident and emergency department with symptoms of reduced appetite, increased fatigue, and mild lower abdominal pain with loose stools and reduced urine output. He had also been complaining of lower back pain for a week. He was originally diagnosed with recto-sigmoid cancer in 2020, which was treated with anterior resection; however, he had a recurrence of cancer a few months ago on CT scan and biopsy. His physical exam was unremarkable, including a normal digital rectal examination. He was afebrile and haemodynamically stable. His blood gas showed metabolic acidosis with a pH of 7.24 and a bicarbonate level of 14 mmol/L. His blood tests showed hyperkalaemia and stage 3 acute kidney injury (AKI) according to KDIGO criteria [8] (Table 1). The CT scan of the abdomen and pelvis revealed new bilateral renal pelvicalyceal dilatation. On the right, this was due to a low right rectal/mesorectal recurrence. On the left, it arose from a lesion in the descending colon as it passed anterior to the iliac vessels. There was no free gas present, and no small or large bowel dilatation. The lung bases were clear (Figures 1-3).

**Table 1:** Lab values

Parameters	Initial Value	Value After Conservative Management	Value After Bilateral Nephrostomies	Reference Range
Urea (mmol/L)	36.7	26.9	4.0	2.5 - 7.1
Creatinine (μmol/L)	1302	826	97	60 - 110
Potassium (K⁺) (mmol/L)	6.1	4.7	3.8	3.5 - 5.3
eGFR (ml/min/1.73m²)	3	5	63	>60

The patient was admitted to the ITU with a diagnosis of AKI secondary to bilateral ureteric obstruction due to colorectal carcinoma. He was catheterised and had a low urine output of 50 mL. He was initially treated with calcium gluconate, salbutamol, dextrose/insulin infusion, sodium bicarbonate, and sodium zirconium cyclosilicate. After treatment, his blood tests showed minimal improvement, but his eGFR and urine output still remained poor. Hence, on the second day, bilateral nephrostomies were performed, and his urine output started to improve, as shown in Table 1. By the fourth day, he was producing good urine output; additionally, as his blood tests showed further improvement, he was discharged to the urology ward the same day. On the 12th day, the bilateral nephrostomy tubes were replaced with antegrade bilateral ureteric stents. On the 14th day, he was discharged home. 

## Discussion

This case report aims to highlight that colorectal cancer is an uncommon cause of bilateral ureteric obstruction. In a case review series involving 50 patients with bilateral ureteric obstruction, 38 cases were found to be caused by malignancy, while the remaining 12 were benign [6]. Amongst the 38 malignant cases, cervical cancer was the most common, with 11 cases reported, while only five patients presented with colon cancer [6]. This emphasizes the relative rarity of colon cancer as a cause of bilateral ureteric obstruction.

BPH is recognized as the most common cause of obstructive uropathy. In the female population, the most common cause is a pelvic mass, while ureteric stones are the predominant factor among middle-aged adults and patients having a single kidney [3]. Our 81-year-old patient suffered from obstructive uropathy due to colorectal carcinoma, which is a relatively uncommon scenario.

Patients with ureteric obstruction usually have abdominal and flank pain and anuria [4, 6]. Associated symptoms of constipation, nausea/vomiting, and diarrhoea can also occur due to underlying bowel obstruction or colonic cancer [4]. Any patient with suspected ureteric obstruction should have a complete physical exam, including a digital rectal examination and abdominal examination. A distended bladder should point the physician toward the possibility of urinary retention [4].

The initial tests should include blood gases and blood tests, including a full blood count, serum creatinine, urea, and electrolytes [1]. The best modality for diagnosing post-renal AKI is an abdominal ultrasound scan or CT scan to look for hydronephrosis [1, 5]. In our case, a CT scan was used to detect hydronephrosis.

The initial management of post-renal AKI is the same as for pre-renal and intrarenal AKI, which consists of supportive treatment and correcting electrolyte imbalances. However, unlike other types of AKI, the definitive management of post-renal AKI is to relieve the obstruction [1].

Hyperkalaemia is treated by administering calcium gluconate, salbutamol, dextrose/insulin infusion, and sodium bicarbonate, as well as ion exchange resins [9]. Our patient was prescribed calcium gluconate, salbutamol, dextrose/insulin infusion, sodium bicarbonate, and the ion exchange resin sodium zirconium cyclosilicate. The patient can be commenced on renal replacement therapy (RRT) if conservative management fails [1]. Our patient did not require RRT, as his hyperkalaemia responded successfully to conservative treatment.

For treating ureteral obstruction, percutaneous nephrostomy (PCN) and double-J stents remain the most popular methods [1, 5]. In our case, PCN was used to relieve the ureteric obstruction.

Colorectal cancers causing ureteric obstructions are rarely surgically removed due to extensive local spread or metastasis. Usually, a conservative or palliative treatment is used [10]. Our patient's cancer wasn’t surgically removed due to its extensive spread.

## Conclusions

Colorectal cancer is an uncommon cause of bilateral ureteric obstruction. This report, therefore, highlights the importance of considering it as a significant cause of obstructive uropathy and post-renal acute kidney injury. This case reflects an advanced stage of recurrent colorectal malignancy causing ureteric obstruction. A CT scan is a good diagnostic tool and can be used to diagnose the obstruction and its cause. Colorectal cancers causing these obstructions are usually not surgically excised due to extensive spread and are treated palliatively. Better recovery of renal function is associated with early diagnosis and timely relief of obstruction.
